# *Bifidobacterium animalis *AHC7 protects against pathogen-induced NF-κB activation *in vivo *

**DOI:** 10.1186/1471-2172-11-63

**Published:** 2010-12-22

**Authors:** David O'Mahony, Sharon Murphy, Thomas Boileau, JeanSoon Park, Frances O'Brien, David Groeger, Patrycja Konieczna, Mario Ziegler, Paul Scully, Fergus Shanahan, Barry Kiely, Liam O'Mahony

**Affiliations:** 1 Alimentary Health Ltd., National University of Ireland, Cork, Ireland; 2 Procter & Gamble, Lewisburg, Ohio, USA; 3 Alimentary Pharmabiotic Centre, National University of Ireland, Cork, Ireland; 4 Swiss Institute of Allergy and Asthma Research, University of Zurich, Davos, Switzerland

## Abstract

**Background:**

Bifidobacteria and lactobacilli are among the early and important colonizers of the gastrointestinal tract and are generally considered to be part of a normal, healthy microbiota. It is believed that specific strains within the microbiota can influence host immune-reactivity and may play a role in protection from infection and aberrant inflammatory activity. One such strain, *Bifidobacterium animalis *AHC7, has been previously shown to protect against *Salmonella typhimurium *infection in mice and helps resolve acute idiopathic diarrhea in dogs. The aim of this study was to investigate the potential molecular and cellular mechanisms underpinning the *Bifidobacterium animalis *AHC7 protective effect.

**Results:**

Following 4 hours of infection with *Salmonella typhimurium*, NF-κB activation was significantly elevated *in vivo *in placebo and *Enterococcus faecium*-fed animals while *Bifidobacterium animalis *AHC7 consumption significantly attenuated the NF-κB response. *In vitro *anti-CD3/CD28 stimulated Peyer's patch cells secreted significantly less TNF-α and IFN-γ following *Bifidobacterium animalis *AHC7 consumption. Stimulated cells released more IL-12p70 but this difference did not reach statistical significance. No alteration in mucosal IL-6, IL-10 or MCP-1 levels were observed. No statistically significant change in the cytokine profile of mesenteric lymph node cells was noted. *In vitro*, *Bifidobacterium animalis *AHC7 was bound by dendritic cells and induced secretion of both IL-10 and IL-12p70. In addition, co-culture of CD4+ T cells with *Bifidobacterium animalis *AHC7-stimulated dendritic cells resulted in a significant increase in CD25+Foxp3+ T cell numbers.

**Conclusion:**

*Bifidobacterium animalis *AHC7 exerts an anti-inflammatory effect via the attenuation of pro-inflammatory transcription factor activation in response to an infectious insult associated with modulation of pro-inflammatory cytokine production within the mucosa. The cellular mechanism underpinning *Bifidobacterium animalis *AHC7 mediated attenuation of NF-κB activation may include recognition of the bacterium by dendritic cells and induction of CD25+Foxp3+ T cells.

## Background

It is becoming increasingly clear that the microbiota condition and prime immunological function with an unexpected level of interdependence between bacteria and the immune system [1]. Accumulating evidence suggests that certain bacterial strains provide protective signals while other bacterial strains stimulate aggressive and damaging immune responses [2-5]. In other words, the activity of the mammalian immune system seems to be governed by the balance between symbiotic and potentially pathogenic factors derived from our microbial inhabitants. This raises the possibility that dysbiosis can lead to inappropriate inflammatory responses while on the other hand certain well selected anti-inflammatory microbes may protect against aberrant inflammatory activity.

The most important aspect of immunological function is the ability to protect against infectious microbes. The host response to infection requires innate and acquired cellular and humoral immune reactions, designed to limit spread of the offending organism and to restore organ homeostasis [6]. However, to limit the aggressiveness of collateral damage to host tissues, a range of regulatory constraints may be activated, such as induction of T regulatory cells [7]. A successful immune response is characterized by the efficient elimination of the pathogenic organism with minimal inflammatory damage to the host and the associated inflammatory cascades which may promote inflammatory disease. Innate pro-inflammatory signaling in response to microbial exposure is mediated by the activation of transcription factors, such as NF-κB, resulting in expression of a battery of effector molecules contributing to host defense and inflammation [8]. A number of bacterial products have been identified which directly block activation of the NF-κB pathway in epithelial cells via a range of novel mechanisms including the blockade of Iκ-B poly-ubiquination by non-pathogenic *Salmonella *strains or the enhancement of NF-κB export from the nucleus by *Bacteroides thetaiotaomicron *[9,10]. In addition, non-pathogenic microbes such as *Bifidobacterium infantis *35624 have been demonstrated to limit excessive NF-κB activation via the induction of T regulatory cells [11].

Interest in the deliberate administration of microbes, or microbial metabolites, for the treatment of aberrant inflammatory activity associated with an exuberant immune response to pathogens is gaining momentum. The typical microbes which are currently being examined include *Bifidobacteria*, *Lactobacilli*, non-pathogenic *E. coli *and *Bacteroides *strains [12-17]. The protective effects associated with these microbes are probably mediated by multiple mechanisms involving epithelial cells, dendritic cells and T cells. One such organism, *Bifidobacterium animalis *AHC7 (*B. animalis *AHC7), has been previously shown to protect against *Salmonella typhimurium *infection in murine models and helps resolve acute diarrhea in dogs [18,19]. However, the host immunological molecular events contributing to this protective mechanism have not been described. Therefore, we have examined activation of the pro-inflammatory transcription factor NF-κB in animals pretreated with *B. animalis *AHC7 or *E. faecium *SF68. *E. faecium *SF68 has been previously described as a probiotic organism with immunomodulatory activity and we used this bacterial strain as a comparator microbe for *B. animalis *AHC7 [20,21]. In addition, we have examined the influence of *B. animalis *AHC7 on dendritic cell activation and T cell polarization. Our data suggests that the *B. animalis *AHC7 protective effect is associated with modulation of NF-κB activity *in vivo *while *in vitro *studies demonstrate that dendritic cell recognition of this bacterium is associated with induction of CD25+Foxp3+ T cells.

## Results

### B. animalis AHC7 suppresses NF-κB activation in vivo

**Figure 1:**
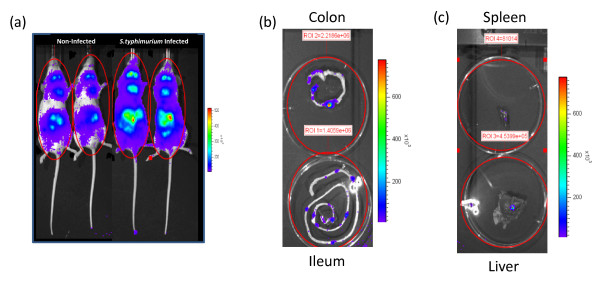
**Biophotonic imaging of *S. typhimurium *induced NF-κB activation**. Following 4 hours of infection with *S. typhimurium *in mice fed control diets, whole body biophotonic imaging revealed a significant increase in NF-κB activation. Two representative animals are illustrated (a). Isolated gastrointestinal tissue from *S. typhimurium*-infected animals displays enhanced NF-κB activation within the ileum and colon (b). Isolated spleen and liver tissue also display NF-kB activity following *S. typhimurium *infection (c). The region of interest (ROI) is shown with the photons/sec/cm^2 ^value for representative ROI included.

**Figure 2:**
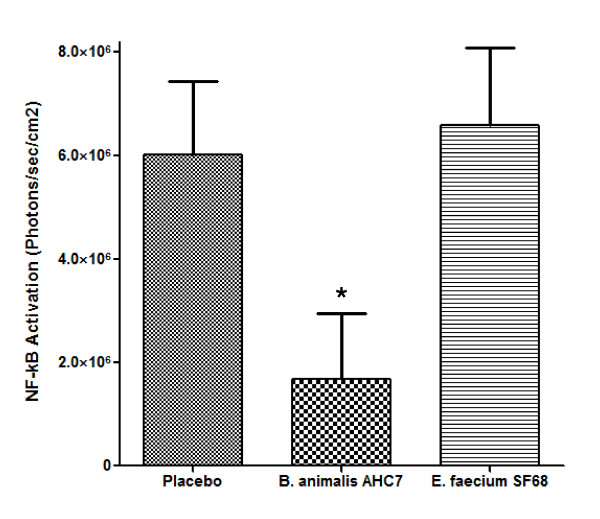
***B. animalis *AHC7 attenuates *S. typhimurium *induced NF-κB activation**. Mice consumed placebo, *B. animalis *AHC7 or *E. faecium *for 3 weeks prior to *S. typhimurium *infection. Biophotonic imaging was performed on all animals at time 0 and 4 hours following infection. The increase in bioluminescence (i.e. NF-κB activation) from baseline (time 0) to 4 hours is illustrated for each group. *p < 0.05 versus placebo (n = 10 animals per group).

Infection of mice with *S. typhimurium *results in potent activation of NF-κB systemically after 4 hours (Figure 1a). Representative animals were dissected and individual organs re-imaged for localisation of *in vivo *NF-κB activity (Figure 1b). Isolated ileum displayed specific areas of activation which macroscopically co-localized with Peyer's patches. Within the colon, a different pattern was observed with a single foci of high activity associated with a lower level of activation at the proximal and distal segments. Isolated spleen and liver also displayed elevated NF-κB activity following 4 hours of *S. typhimurium *infection. Placebo-fed animals displayed a significant up-regulation of NF-κB whole body activity after 4 hours infection (Figure 2). Pre-feeding with *B. animalis *AHC7 significantly attenuated the activation of this pro-inflammatory transcription factor. In order to determine if the attenuation of NF-κB activity was bacterium-strain specific, additional mice were pre-fed *E. faecium *prior to *S. typhimurium *infection. Up-regulation of NF-κB activity was similar in the placebo-fed animals and *E. faecium*-fed animals (Figure 2).

### B. animalis AHC7 mucosal immunoregulatory activity

**Figure 3:**
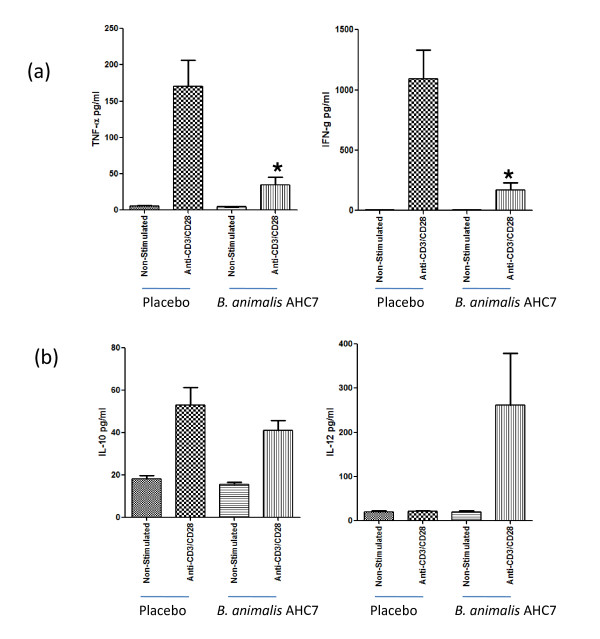
**Peyer's patch cytokine secretion**. Isolated Peyer's patch cells were cultured *in vitro *with or without anti-CD3/CD28 stimulation. Secretion of TNF-α and IFN-γ was significantly reduced in animals pre-fed *B. animalis *AHC7 (a). Secretion of IL-10 was unaltered between the groups while a non-statistically significant increase in IL-12 secretion was observed (b). *p < 0.05 versus placebo (n = 10 animals per group).

In order to determine if lymphocytes within the mucosa could be influenced by *B. animalis *AHC7 consumption, healthy mice were administered the bacterial strain or placebo for 3 weeks. Peyer's patch lymphocytes displayed an altered cytokine secretion profile following *in vitro *stimulation. Anti-CD3/CD28 antibody stimulation of Peyer's patch cells from *B. animalis *AHC7 fed animals secreted significant lower levels of IFN-γ and TNF-α (Figure 3a). In contrast, secretion of IL-10 was unaltered while IL-12p70 levels were increased (p = 0.07) compared to placebo-fed animals (Figure 3b). No differences were noted for IL-6 or MCP-1 levels (data not shown) or for the un-stimulated cultures. In addition, no alteration in anti-CD3/CD28 stimulated mesenteric lymph node secretion of IL-6, IL-10, IL-12p70, TNF-α, IFN-γ or MCP-1 was observed for *B. animalis *AHC7-fed animals compared to placebo-fed controls (data not shown).

### B. animalis AHC7 interaction with dendritic cells

**Figure 4:**
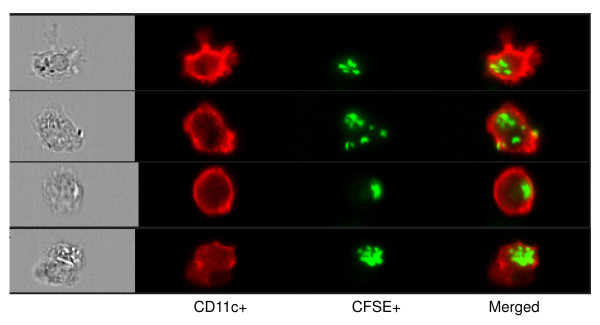
**Dendritic Cells Bind *B. animalis *AHC7**. Representative pictures are shown which illustrate binding of CFSE-labeled *B. animalis *AHC7 (green) to CD11c+ monocyte derived dendritic cells (red).

**Figure 5:**
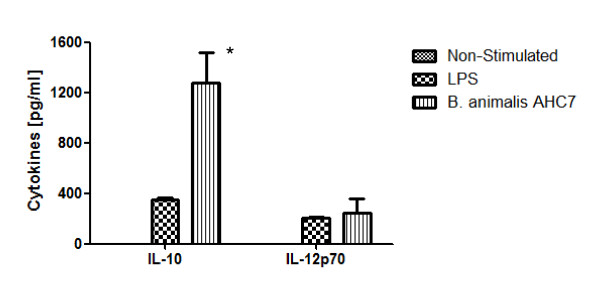
**Dendritic Cells Secrete IL-10 and IL-12p70 in Response to *B. animalis *AHC7**. MDDCs co-incubated with *B. animalis *AHC7 or LPS for 24 hours secrete significant levels of IL-10 and IL-12p70. Results represent the mean of three independent experiments. *p < 0.05 versus LPS-stimulated MDDCs.

Human monocyte derived dendritic cells (MDDCs) were co-incubated with CFSE-labelled *B. animalis *AHC7 *in vitro *and imaged for evidence of bacterial binding. CFSE labeled *B. animalis *AHC7 was easily visualized on the cell surface of CD11c+ MDDCs indicating efficient binding of this bacterium by human myeloid dendritic cells (Figure 4). In addition, MDDCs secreted IL-10 and IL-12p70 in response to *B. animalis *AHC7 (Figure 5). Indeed, IL-10 secretion in response to this microbe was significantly greater than the MDDC response to LPS while the level of IL-12p70 secretion was equivalent to that induced by LPS.

### B. animalis AHC7 conditioned dendritic cells influence T cell polarisation

**Figure 6:**
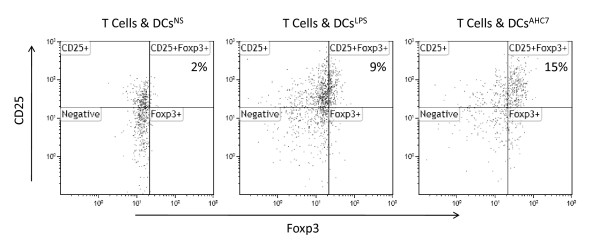
***B. animalis *AHC7 stimulated MDDCs induce CD25+Foxp3+ T Cells**. Following co-incubation with MDDCs, CD4+ T cells were stained for CD25 and Foxp3. *B. animalis *AHC7 stimulated MDDCs induced more CD25+Foxp3+ T cells than LPS stimulated or non-stimulated MDDCs. Representative results from three independent experiments are illustrated.

Following co-incubation with *B. animalis *AHC7 for 4 hours, MDDCs were extensively washed and co-incubated with autologous CD4+ T cells for 5 days. T cells were re-stimulated with anti-CD3/CD28 antibodies for 2 days and transcription factor levels quantified by flow cytometry. *B. animalis *AHC7-stimulated MDDCs induced significantly more CD25+Foxp3+ T cells compared to non-stimulated MDDCs or LPS stimulated MDDCs (Figure 6).

## Discussion

This report illustrates, at a cellular and molecular level, the impact of the commensal microbiota on host immune defense and immune homeostasis. The deliberate consumption of one commensal organism, *B. animalis *AHC7, resulted in the attenuation of NF-κB activation within mice infected with a pro-inflammatory translocating microbe, *S. typhimurium*. In addition, *B. animalis *AHC7 consumption was associated with modulation of cytokine signaling within the mucosa of healthy animals. *In vitro*, dendritic cells bound *B. animalis *AHC7, secreted IL-10 and IL-12p70, and dendritic cells stimulated with *B. animalis *AHC7 enhanced Foxp3 expression in naïve T cells.

Infection by microbes such as *Salmonella *species results in severe immunopathology characterized by loss of intestinal barrier function, tissue cell death and loss of function, fever and ultimately can lead to death of the host [22]. The pathology is not only a direct effect of the microbe itself but is a consequence of the inflammatory response induced by the presence of the organism. Therefore *Salmonella *infection of mice is a good model system to examine the regulatory mechanisms which protect against excessive pro-inflammatory responses to a range of pro-inflammatory stimuli and not only *Salmonella *itself. The resident microbiota can aid in the protection against aberrant inflammatory activity as suggested by studies in germ-free mice where infection with *S. typhimurium *results in colitis which was not observed in conventionally colonized animals [23]. In addition, specific commensal organisms such as *B. animalis *AHC7 protect against *S. typhimurium *infection and disease severity [19]. While *B. animalis *AHC7 may directly antagonize *Salmonella *within the gastrointestinal tract, this is unlikely to be the primary mechanism as other commensal organisms which were significantly more effective than *B. animalis *AHC7 at killing *S. typhimurium in vitro*, did not protect against infection *in vivo *[19]. These studies suggest that the biological activity of certain commensal microbes is exerted well beyond a direct influence on the microbiota within the gastrointestinal tract. Therefore, we investigated whether *B. animalis *AHC7 could exert an effect on the host via regulation of the pro-inflammatory response as a potential mechanism underpinning its protective effect.

*S. typhimurium *infects the host via intestinal epithelial cells and dendritic cells. In addition, Salmonella infects via M cells which transport the bacterium to underlying Peyer's patches for immunological processing [24]. Infection of the Peyer's patches leads to recruitment of a large number of pro-inflammatory infiltrating leukocytes which further aggravate intestinal inflammation and promote systemic dissemination of the pathogen. *B. animalis *AHC7 consumption leads to an altered cytokine profile within Peyer's patches which may protect against excessive inflammation. Release of TNF-α and IFN-γ by stimulated Peyer's patch lymphocytes *in vitro *is significantly reduced. In contrast, release of the Th1 cytokine IL-12 is enhanced with no alteration in IL-6, IL-10 or MCP-1 levels. Interpretation of these results is complex as IFN-γ and IL-12 are both considered to be Th1 cytokines. IL-12p70 is released by cells of the innate immune system, such as dendritic cells, while IFN-γ is secreted by T cells suggesting that *B. animalis *AHC7 consumption may differentially regulate dendritic cell and T cell cytokine production. *In vitro*, *B. animalis *stimulated dendritic cells secrete IL-12 and therefore the increased IL-12 release by Peyer's patch cells may be dendritic cell derived. Regardless of the mechanism, it is clear that *in vitro *stimulated cytokine responses from Peyer's patch cells are modulated by *B. animalis *AHC7 consumption and it is likely that these altered responses contribute to the anti-inflammatory effect observed in the *Salmonella *model. In particular, reduced secretion of TNF-α, which is a key pro-inflammatory cytokine, would reduce the inflammatory burden following *S. typhimurium *infection.

Innate immune activation to *Salmonella *is mediated via pattern recognition receptors, such as TLR-5, which rapidly up-regulate NF-κB activity [25]. Both mucosal and systemic NF-κB activation in response to *Salmonella *infection was noted suggesting that widespread activation of the innate immune system occurs rapidly and this response is modulated by *B. animalis *AHC7 consumption. The molecular basis for this inhibitory activity is not known and may involve induction of suppressor molecules, induction of regulatory cells (such as Tregs), down-regulation of TLR expression and/or activity and enhancement of the mucosal immunological barrier. Previously we have shown that increased numbers of CD25+Foxp3+ T cells can reduce NF-κB activation *in vivo *[11]. We did not assess CD25+Foxp3+ T cell polarization in *B. animalis *AHC7- fed animals but the *in vitro *co-culture model using *B. animalis *conditioned dendritic cells clearly demonstrated that this bacterium can induce a dendritic cell response which induces CD25+Foxp3+ T cells. However, it remains to be determined if this mechanism is responsible for the *B. animalis *AHC7 anti-inflammatory effect *in vivo*.

## Conclusion

NF-κB is a key pro-inflammatory transcription factor and improved regulation of NF-κB is an important therapeutic target in a wide range of pro-inflammatory states, including sepsis [26]. This report supports the clinical evaluation of appropriately selected probiotic/commensal micro-organisms, such as *B. animalis *AHC7, for the promotion of regulatory mechanisms *in vivo *which limit aberrant pro-inflammatory activity. However, it is clear from this study and others that not all commensal microbes modulate host immunological activity in the same way and the defining features of potent anti-inflammatory microbes remain to be described.

## Methods

### Bacterial strains

*B. animalis *AHC7 was routinely cultured anaerobically for 48 hours in deMann, Ragosa and Sharpe medium, MRS (Oxoid, Basingstoke, UK) supplemented with 0.05% cysteine (Sigma, Dublin, Ireland). *Enterococcus faecium *SF68 was routinely cultured aerobically at 37°C for 24 hours in on tryptic soya broth, TSB (Oxoid). Freeze-dried powders of these strains were generated for use in subsequent animal studies. *Salmonella typhimurium *UK1 was generously provided by Prof. Roy Curtiss III (Washington University, USA) and was routinely cultured aerobically at 37°C for 24 hours in TSB (Oxoid).

### Murine studies

NF-κB^lux ^transgenic mice on a Balb/c background were obtained from Charles River Laboratories (MA, USA) and bred in-house for salmonella infection studies. Mice were housed under barrier maintained conditions within the biological services unit, University College Cork (UCC). All animal experiments were approved by the UCC animal ethics committee and experimental procedures were conducted under appropriate license from the Irish government. Female NF-κB^lux ^mice were administered freeze-dried *B. animalis *AHC7 or *E. faecium *SF68 in their water supply at approximately 5 × 10^8 ^CFU/day or cryoprotectant carrier (n = 10 per group) for 3 weeks prior to a single oral challenge with 1 × 10^7 ^*S. typhimurium*. NF-κB activation was visualized at time 0 and 4 hours following infection using the protocol as previously described [11,27]. Briefly, D-luciferin (120 mg/kg; Biothema AB, Handen, Sweden) was injected i.p. and immediately anaesthetized mice were placed in a ventral recumbent position in an In Vivo Imaging System (IVIS) chamber (Xenogen, Alameda, USA) and imaged continuously for 5 minutes with a medium sensitivity setting starting 2 minutes after the injection of D-luciferin. Photons were quantified using Living Image software (Xenogen) and the luciferase activity quantified as the amount of light emitted per second per cm^2 ^from the animal. Following imaging, all mice were humanely euthanized.

Wild-type Balb/c mice were obtained from Harlan (Oxon, UK) and bred in-house for bifidobacterial feeding. Female Balb/c mice (n = 8 per group) received either freeze-dried *B. animalis *AHC7 or placebo (cryoprotectant carrier) administered on a daily basis in their drinking water. Each animal received an approximate dose of 5 × 10^8 ^CFU/day. Drinking water bottles were changed every day with fresh probiotic freeze-dried powders as previously described [19,28]. After 3 weeks of treatment the mice were humanely euthanized followed by isolation of Peyer's patches and mesenteric lymph nodes.

### In vitro Culture of Mucosal Cells

Single cell suspensions from Peyer's patches or mesenteric lymph nodes from Balb/c mice were cultured *in vitro *for 48 hours with anti-CD3/anti-CD28 antibodies (BD Biosciences, Oxford, UK) or remained non-stimulated. Supernatants were harvested for cytokine analysis and stored at -80°C until quantification by multiplex profiling. IL-6, TNF-α, IL-10, IL-12p70, IFN-γ and MCP-1 cytokine levels were quantified using cytometric bead arrays (BD).

### Dendritic Cell Isolation and Culture

Human CD14+ monocytes were isolated from peripheral blood using antibody labeling and magnetic separation (Miltenyi, Gladbach, Germany) and cultured in the presence of IL-4 (200 ng/ml, gift from Novartis, Basel, Switzerland) and GM-CSF (1,000 Units/ml, Peprotech, Hamburg, Germany) for 5 days to generate CD11c+ monocyte derived dendritic cells (MDDCs). 5 × 10^5 ^MDDCs were incubated with 1 × 10^7 ^*B. animalis *AHC7 labeled with carboxyfluorescein succinimidyl ester (CFSE, Invitrogen, Carlsbad, USA). After two hours incubation, MDDCs were stained with anti-human CD11c-PE-Cy5 (BD) and cells were visualized using the Image Stream X system (Amnis Corporation, Seattle, USA) and analyzed with IDEAS software (Amnis Corporation). In addition, non-CFSE stained *B. animalis *AHC7, or LPS (Sigma), were used to stimulate MDDCs for 24 hours and cytokine levels in the culture supernatants were measured by the multiplex Luminex platform.

### MDDC-T Cell Co-Culture

MDDCs were isolated as above and autologous CD4+ T cells were isolated using antibody binding and magnetic separation (Miltenyi). MDDCs were stimulated with *B. animalis *AHC7, LPS or remained non-stimulated for four hours and washed four times to remove un-bound bacteria. MDDCs were co-incubated with autologous CD4+ T cells for five days (1:20 ratio) in FCS-free medium followed by re-stimulation with anti-CD3/CD28 antibodies for two days. Flow cytometric quantification of CD4+CD25+Foxp3+ T cells (all antibodies from eBioscience, Frankfurt, Germany) was performed on a Gallios flow cytometer (Beckman Coulter) and the data was analysed using Kaluza software (Beckman Coulter).

### Statistics

Two way-ANOVA with Bonferroni's Post-test was used to determine statistical significance between treatment groups for NF-κB activation. The differences in cytokine levels between groups was evaluated using a Mann-Whitney t-test. All data were expressed as arithmetic mean±SEM. The level of statistically significance was set at p < 0.05. All statistical evaluations were performed using the statistical software package GraphPad Prism 4.03 (GraphPad Software, Inc., San Diego, CA, USA).

## Authors' contributions

DOM, FOB, DG and PS performed the *Salmonella *infection and non-infected murine experiments and the associated cytokine analysis. SM performed the *in vivo *NF-κB measurements. TB, FS and BK contributed to the design and analysis of all experimental data. PK performed the dendritic cell imaging studies while MZ performed the dendritic cell-T cell co-cultures. JSP contributed to the data analysis and preparation of the manuscript while LOM conceived the studies, contributed to the study design, data interpretation and manuscript preparation. All authors read and approved the final version of this manuscript.
